# Identification of HLA-A2-Restricted Mutant Epitopes from Neoantigens of Esophageal Squamous Cell Carcinoma

**DOI:** 10.3390/vaccines9101118

**Published:** 2021-10-01

**Authors:** Zhiwei Wang, Ling Ran, Chunxia Chen, Ranran Shi, Yu Dong, Yubing Li, Xiuman Zhou, Yuanming Qi, Pingping Zhu, Yanfeng Gao, Yahong Wu

**Affiliations:** 1 School of Life Sciences, Zhengzhou University, Zhengzhou 450001, China; 202011161010131@gs.zzu.edu.cn (Z.W.); 18039215962@163.com (L.R.); chunxiachen@gs.zzu.edu.cn (C.C.); srr@gs.zzu.edu.cn (R.S.); 16637144372@163.com (Y.D.); liyubing7788@gs.zzu.edu.cn (Y.L.); zhouxm36@mail.sysu.edu.cn (X.Z.); qym@zzu.edu.cn (Y.Q.); zhup@zzu.edu.cn (P.Z.); 2 International Joint Laboratory for Protein and Peptide Drugs of Henan Province, Zhengzhou University, Zhengzhou 450001, China; 3 State Key Laboratory of Esophageal Cancer Prevention & Treatment, Zhengzhou University, Zhengzhou 450052, China; 4 School of Pharmaceutical Sciences (Shenzhen), Sun Yat-sen University, Shenzhen 510080, China

**Keywords:** esophageal squamous cell carcinoma, cytotoxic T lymphocytes, mutant peptide, neoantigen, immunotherapy

## Abstract

Esophageal squamous cell carcinoma (ESCC), one of the deadliest gastrointestinal cancers, has had limited effective therapeutic strategies up to now. Accumulating evidence suggests that effective immunotherapy in cancer patients has been associated with T cells responsive to mutant peptides derived from neoantigens. Here, we selected 35 human leukocyte antigen-A2 (HLA-A2)-restricted mutant (MUT) peptides stemmed from neoantigens of ESCC. Among them, seven mutant peptides had potent binding affinity to HLA-A*0201 molecules and could form a stable peptide/HLA-A*0201 complex. Three mutant peptides (TP53-R267P, NFE2L2-D13N, and PCLO-E4090Q) of those were immunogenic and could induce the cytotoxic T lymphocytes (CTLs) recognizing mutant peptides presented on transfected cells in an HLA-A2-restricted and MUT peptide-specific manner. In addition, the CTL response in immunized HLA-A2.1/K^b^ transgenic (Tg) mice was enhanced by coupling MUT peptides to peptide WH, a peptide delivery carrier targeting Clec9a^+^ DCs. Then, the possible binding model conversions between the WT and MUT candidate peptides were analyzed by docking with the pockets of HLA-A*0201 molecule. We therefore propose a novel strategy and epitopes for immunotherapy of ESCC based on neoantigens.

## 1. Introduction

Esophageal cancer (EC) ranks fifth in terms of incidence and fourth in mortality in China, where numbers of new cases and cancer deaths account for more than half of the cases occurring worldwide [1,2]. The categories of esophageal cancer are classified as adenocarcinoma (EAC) or squamous cell carcinoma (ESCC, main incidence type in China) by histopathology [3]. The 5-year survival of esophageal carcinoma patients is extremely poor owing to the disappointing fact that no promising strategy has been established for patients other than the standard therapies of surgery, chemotherapy, or radiotherapy [4]. More than half of EC patients present with advanced, metastatic, or unresectable cancer. For this majority of patients who are unsuitable for surgery, the standard treatments are effective palliative interventions to relieve dysphagia, improve quality of life, and prolong survival [5]. Therefore, it is urgent to develop a new approach for ESCC patients who suffer from advanced, metastatic, or unresectable cancer.

Immune checkpoint blockade, an immunotherapy targeting immune checkpoints, has shown conclusive proof of objective response [6,7]. However, the strong clinical response of immune checkpoint blockade only exists in certain patients [8,9,10]. Recent research has revealed that tumor mutational burden (TMB) is an emerging biomarker for identifying patients who will derive a strong objective response to checkpoint blockade [11,12]. TMB is significantly related to tumor mutant antigen burden, which is the important target of checkpoint blockade therapy. Thus, the key to tumor immunotherapy is to find the immunogenic mutant peptides from among tumor mutant antigens and then to activate the tumor-specific T lymphocytes more effectively.

Fortunately, high-throughput sequencing of 356 ESCC tumor samples has been able to define the mutant antigen landscape of ESCC in recent studies [13,14,15]. Somatic mutations from several genes (such as TP53, NOTCH1, NFE2L2, etc.) were identified in ESCC. Whether these mutant antigens could be served as immunotherapy targets remains unclear. To investigate whether there are new T cell responsive epitopes (especially epitopes restricted to HLA-A2, which is commonly seen among the Chinese population) that are produced according to these mutations, we first exploited the mutant antigen in ESCC reported in these studies to predict HLA-A2-restricted MUT peptides using the online programs NetCTL-1.2, SYFPEITHI and IEDB. To select mutant peptides that were high-affinity and could form stable complexes with HLA-A2 molecules, T2A2 cell binding assays and peptide/MHC complexes stability assays were performed. Subsequently, we utilized peripheral blood mononuclear cells (PBMCs) of HLA-A2^+^ healthy donors to evaluate the capability of mutant peptides to stimulate MUT peptide-specific and HLA-A2-restricted CTL response in vitro. Additionally, the same tests were performed by utilizing HLA-A2.1/K^b^ Tg mice in vivo.

## 2. Materials and Methods

### 2.1. Peptides

Researchers have identified ESCC with a high mutant antigen load using whole exome sequencing (WES) [13,14,15]. To predict neoepitopes, mutant antigens of 356 ESCC tumor samples were analyzed and selected. Then, the HLA-A2-restricted MUT peptides stemmed from mutant antigen of ESCC, which had appropriate prediction scores meeting all the three prediction programs score thresholds (NetCTL-1.2 score > 0.85, SYFPEITHI score > 20, and IEDB rank < 3.5), were predicted by the T cell epitope prediction program NetCTL-1.2 (http://www.cbs.dtu.dk/services/NetCTL/, accessed on 25 March 2019), SYFPEITHI (http://www.syfpeithi.de/bin/MHCServer.dll/EpitopePrediction.htm, accessed on 25 March 2019), and IEDB (http://tools.immuneepitope.org/mhci/, accessed on 25 March 2019). Mutant peptides and their corresponding wild-type peptides were synthesized and purified (> 95%) by Fmoc solid phase synthesis and reverse-phase high-performance liquid chromatography, and the molecular weight of these peptides was confirmed by electrospray ionization mass spectrometry (ESI–MS). Cyclooxygenase-2(COX-2)_321-329_ (ILIGETIKI) was served as positive control in the T2A2 cell-binding assay. Wild-type (WT) peptides corresponding to their MUT peptides were used in in vitro assays. WH (WPRFHSSVFHTH)-MUT peptides and WH peptide linking with MUT peptide by GGG linker were used in in vivo assays [16]. All the purified peptides were dissolved in 2% DMSO-PBS buffer (PH 7.2, v:v) at a concentration of 10 mg/mL and reserved at −80 °C.

### 2.2. Cell Lines, Peripheral Blood Mononuclear CELLS (PBMCs), and Animal

T2A2 cells (TAP-deficient HLA-A2 molecules overexpressing, cultured in IMDM medium), human esophageal squamous carcinoma tumor cell line KYSE140 (HLA-A2-positive (HLA-A2^+^), cultured in RPMI 1640 medium), KYSE150 (HLA-A2-negative (HLA-A2^−^), cultured in RPMI 1640 medium), and HEK-293T (cultured in DMEM medium) were purchased from American Type Culture Collection (ATCC, Manassas, VA, USA).

PBMCs were isolated from HLA-A2^+^ healthy donors’ peripheral blood samples, obtained from Henan Red Cross Blood Center with the approval of the Institutional Ethics Review Board, with approval number ZZUIRB2020-54.

HLA-A2.1/K^b^ Tg mice (8 weeks) were bred and maintained under specific pathogen-free (SPF) conditions in the Laboratory Animal Center, School of Life Sciences, Zhengzhou University. All the research was performed under the approval of the Ethics Committee of Zhengzhou University or the Institutional Animal Care and Use Committees of Zhengzhou University.

### 2.3. Antibodies

For the T2A2 cell-binding assay, monoclonal antibody HLA-A2 PE-Cyanine7 (clone: BB7.2, # 343314) was purchased from BioLegend (San Diego, CA, USA). For intracellular cytokine staining assay, monoclonal antibody anti-human CD3 eflour450 (clone: OKT3, #48-0037), anti-human CD8α PerCP-eFlour 710 (clone: SK1, #46-0087), anti-human IFN-γ PE (clone: 4S.B3, #12-7319), anti-human granzyme B PE (clone: GB11, #12-8899), anti-mouse CD3 PerCP-eFlour 710 (clone: 17A2, #46-0032), anti-mouse CD8α PE (clone: 53-6.7, #12-0081), anti-mouse IFN-γ APC (clone: XMG1.2, #17-7311), and anti-mouse granzyme B eFlour 450 (clone: NGZB, #48-8898) were purchased from eBioscience (San Diego, CA, USA). To detect the mature cells (DCs), anti-human CD80 PerCP-eFlour 710 (clone: 2D10.4, #46-0809), anti-human CD86 PE (clone: IT2.2, #12-0869), and anti-human HLA-DR APC (clone: LN3, #17-9956) were purchased from eBioscience (USA).

### 2.4. T2A2 Cell-Binding Assay and Stabilization Assay

The binding affinity of neoepitope to the MHC molecule and the stable neoepitope/MHC complex are critical for inducing T cell immune response, so we first performed a T2A2 cell-binding assay and stabilization assay. The T2A2 cell-binding assay was measured as in our previous study [17]. The binding affinity was computed as follows: fluorescence index (FI) = (mean fluorescence intensity (MFI) of peptide group-MFI of the dissolved buffer without peptide group)/MFI of the dissolved buffer without peptide group.

For the stabilization test [18], T2A2 cells were pulsed with 50 μg/mL mutant peptide and 3 μg/mL β2-Microglobulin (β2-M, Merck, Kenilworth, NJ, USA) in IMDM (serum-free) medium for 18 h. After three times washing, free peptides were removed and brefeldin A (BFA, 10 μg/mL, BD Bioscience, San Diego, CA, USA) was added. BFA was removed after 1 h impeding, and the cells were incubated for additional time. Finally, T2A2 was marked with anti-human HLA-A2 PE-Cyanine7 for 30 min, then analyzed by FACS Calibur (BD Bioscience, USA). MFI of 0 h was set as 100%, and the time of half losing MFI of 0 h defined the dissociation complex 50 (DC_50_). DC_50_ was computed as follows: (MFI of 0 h-MFI of (2, 4, or 6 h))/MFI of 0 h × 100%.

### 2.5. Generation of Peptide-Specific T Cells by Autologous DCs

DCs involve and play an important role in initiating CD8^+^ T cell response. Consequently, ten HLA-A2^+^ healthy donors’ PBMCs were isolated, autologous DCs were generated, and CTLs were induced according to the procedure described previously [19,20,21]. In brief, PBMCs were seeded at a density of 5 × 10^6^ cells/well in a 6-well plate, and after 3 h, the non-adherent cells were removed and cryopreserved for future incubation with mature autologous DCs. Differentiation of the adherent cells into dendritic cells was induced by GM-CSF (100 ng/mL, #300-03, PeproTech, Rocky Hill, NJ, USA) and IL-4 (100 ng/mL; #200-04, PeproTech, USA), with replenishment every 2 days. On day 5, maturation of DCs was induced using 100 ng/mL LPS (#L4516, Sigma-Aldrich, St. Louis, MO, USA), and maturation was checked on day 8 by flow cytometry (BD FACSCelesta™) using markers for anti-human HLA-DR, anti-human CD80, and anti-human CD86. Then, mature DCs (1 × 10^5^) were pulsed with each 10 μg /mL individual peptide or no peptides (control) for 2 h at 37 °C. Next, peptide-pulsed DCs were used to prime the previously cryopreserved autologous peripheral blood lymphocytes (PBLs) at a DC/PBL ratio of 1:10 using complete RPMI-1640 media supplemented with 100 IU/mL rhIL-2 (#200-02, PeproTech, USA) and 10 ng/mL rhIL-7 (#200-07, PeproTech, USA). Half of the feeding medium was replenished every 2 days. After 7 days, PBLs were restimulated with freshly pulsed autologous DCs every 7 days. After the third cycle of priming, T cells were collected for functional analyses by intracellular factor staining assay (ICS) and cytotoxic assay (E/T ratios = 12.5:1, 25:1, 50:1).

### 2.6. Induction of Peptide-Specific T Cells from HLA-A2.1/K^b^ Tg Mice

To investigate whether neoepitopes could induce a peptide-specific T cell response *in vivo*, HLA-A2.1/K^b^ Tg mice were used and immunized according to the procedure de-scribed previously [18]. In brief, mutant peptides or WH-mutant peptides (100 μg per mouse) with CpG-ODN 1826 (30 μg per mouse) were injected s.c. in the back of HLA-A2.1/K^b^ Tg mice (five mice per group, randomly grouped) once a week for three weeks. The normal saline (NS) group was immunized s.c. only with CpG ODN 1826 (30 μg/mouse) once a week for three weeks. Five days after the last injection, spleen lymphocytes were isolated and cultured at the concentration of 5 × 10^6^ cells/mL in RPMI 1640 medium. These cells were induced by the same mutant peptides (10 μg/mL) that were used for in vivo stimulation and mIL-2 (30 U/mL) for a week. Intracellular cytokine staining assay (ICS) and cytotoxic assay (E: T ratios = 20:1, 40:1, 80:1) were performed after a week induced in vitro [22].

### 2.7. The Construction of Minigene and Transfection of Tumor Cell Lines

To test whether the mutant peptides TP53-R267P-, NFE2L2-D13N-, and PCLO-E4090Q-induced peptide-specific T cells could lyse tumor cell lines that expressed these peptides, two 51 aa long minigenes encoding the mutant peptides TP53-R267P, NFE2L2-D13N, and PCLO-E4090Q or the corresponding WT sequence were cloned in lentiviral vector pLVX-IRES-ZsGreen1 (purchased from HedgehogBio Science and Technology Lt) [23,24]. Then two vectors were transduced into KYSE140 (HLA-A2^+^) and KYSE150 (HLA-A2^−^) to get KYSE140-MUT (HLA-A2^+^, MUT peptide^+^), KYSE140-WT (HLA-A2^+^, MUT peptide^−^), KYSE150-MUT (HLA-A2^−^, MUT peptide^+^) and KYSE150-WT (HLA-A2^−^, MUT peptide^−^) cells (see Supplementary Materials for details).

### 2.8. Intracellular Cytokine Staining Assay

When target cells were peptide-pulsed T2A2 cells (MUT peptide or its corresponding WT peptide), peptide-specific T cells (from HLA-A2^+^ healthy donors B–F) stimulated by MUT peptides were co-cultured with these cells in the presence of BFA for 5 h. Then T cells were marked by anti-human CD3 and anti-human CD8α monoclonal antibody. Thirty minutes later, cells were fixed by fixation buffer (Thermo Scientific, Waltham, MA, USA) for thirty minutes and washed by permeabilization buffer (Thermo Scientific, USA). Permeabilized T cells were marked by anti-human IFN-γ monoclonal antibody.

As for the transfected cells serving as target cells in the in vitro investigation, peptide-specific T cells (from HLA-A2^+^ healthy donors G–K) induced by MUT peptides were co-cultured with tumor cell lines transfected with WT or MUT minigenes in IMDM medium in the presence of BFA for 5 h. These T cells were marked with anti-human CD3 and anti-human CD8α monoclonal antibody for 30 min prior to fixation and permeabilization. Permeabilized T cells were respectively marked by anti-human IFN-γ or anti-human granzyme B monoclonal antibody.

To test the MUT peptides stimulation of CTL response in vivo, peptide-specific T cells induced from Tg mice were co-cultured with tumor cell lines transfected with WT or MUT minigenes, as described in the in vitro assay. These T cells were marked by surface marker antibodies anti-mouse CD3 and CD8α before fixation and permeabilization. Intracellular cytokines were marked by anti-mouse IFN-γ or anti-mouse granzyme B, respectively, and analyzed by flow cytometry (BD FACSCelesta™, USA).

### 2.9. Cytotoxicity Assay

The cytotoxicity of peptide-specific T cells stimulated by mutant peptides was tested by using Cell Trace Far Red (Thermo Scientific, USA) [25,26,27]. Transfected cells were suspended in PBS (1 × 10^6^ cells/mL) and labelled with Cell Trace Far Red as follows: Control target cells and test target cells were marked by 50 μM and 1 mM Cell Trace Far Red, respectively, at 37 °C for 20 min, protected from light. After incubation, target cells were washed with complete media at 37 °C for 5 min. Equal numbers of control target cells and test target cells were co-cultured with different numbers of peptide-specific T cells (E/T = 12.5:1, 25:1, 50:1, induced from PBMCs of HLA-A2^+^ healthy donors; E/T = 20:1, 40:1, 80:1, induced from spleen lymphocytes of Tg mice) in IMDM medium for 4 h. Subsequently, test target cells were mixed with control target cells in a tube, washed once, and analyzed by flow cytometry. Specific lysis (%) = (numbers of control target cells—numbers of test target cells)/numbers of control target cells × 100% [28,29], where, for tumor cells, the control target cells refer to KYSE140-WT cells marked with 50 μM Cell Trace Far Red, and the test target cell refers to KYSE140-WT or KYSE140-MUT cells marked with 1 mM Cell Trace Far Red, respectively (peptide-specific T cells generated from donors G–K’s PBMCs in vitro or generated from spleen lymphocytes of HLA-A2.1/K^b^ Tg mice in vivo). Additionally, for T2A2 target cells, T2A2 cells unpulsed and marked with 50 μM Cell Trace Far Red were used as the control target cells, while T2A2 cells loaded with MUT peptides or the corresponding WT peptides (50 μg/mL) or T2A2 cells only were marked with 1 mM Cell Trace Far Red and were used as test target cells (peptide-specific T cells stimulated from donors B–F’s PBMCs).

### 2.10. Analyze the Possible Structural Models of the Candidate Peptide and HLA-A*0201 Molecule

To discover the underlying structural mechanisms for distinguish the WT/MUT peptide, structural models of peptide/HLA-A*0201 complexes were docked by MOE (Molecular Operating Environment software). First, we analyzed the possible structures of the WT/MUT peptides by PEP-Fold (http://bioserv.rpbs.univ-paris-diderot.fr/services/PEP-FOLD/, accessed on 21 May 2020) and downloaded all the results of the peptides [30]. Then the data were computationally introduced into MOE software to obtain different clusters, where the clusters that docked at the binding grooves of HLA-A*0201 and with the high marks had priority [31]. Subsequently, the selected peptide structures were docked into HLA-A*0201 (using Protein Data Bank (PDB) ID 5YXN as templates) peptide-binding grooves using MOE. The potential binding sites of the peptides to HLA-A*0201 molecules were labelled.

### 2.11. Statistical Analysis

All data represent means ± SD. Statistical significance was determined by one-tailed Student’s *t*-test. *p* < 0.05, *p* < 0.01, and *p* < 0.001 were considered statistically significant versus the control group.

## 3. Results

### 3.1. Prediction, Synthesis, and Binding Capacity of MUT Peptide Derived from Mutant Antigen of ESCC

To investigate whether these mutant antigens in ESCC could produce mutant peptide (MUT peptides)-specific activity for immunotherapy, we screened common mutant antigens in ESCC and predicted HLA-A*0201-restricted MUT peptides basing on prediction programs NetCTL-1.2, SYFPEITHI, and IEDB. Altogether, 35 MUT peptides met all the threshold values of these three programs (Table 1). The 35 selected MUT peptides were then synthesized and confirmed (Table 2).

Binding affinity of these peptides with HLA-A*0201 molecule and stability of the peptide/HLA-A*0201 complex they formed are the basis for the peptide to elicit a specific immune response. TAP-deficient T2A2 cells were used to evaluate the binding affinity and the stability of the peptide/HLA-A*0201 complex. The results show that 13 of the 35 MUT peptides, such as MUC16-A9832D, SYNE1-V7402L, and TP53-P190L and so on, were high-binding affinity peptides (FI > 1, Table 2), and all other MUT peptides were low binding affinity peptides (FI < 1, Table S1); additionally, 7 of the 13 high-binding affinity MUT peptides formed stable peptide/HLA-A*0201 complexes (DC_50_ > 4 h, Table 2). According to these results, MUT peptides MUC16-A9832D, SYNE1-V7402I, TP53-P190L, TP53-R267P, ABCA13-E1359Q, NFE2L2-D13N, and PCLO-E4090Q were selected to investigate their capability to stimulate CTL response in vitro.

### 3.2. MUT Peptide-Specific T Cells Can Recognize MUT Peptide-Pulsing T2A2 Cells in MUT Peptide-Specific Manner In Vitro

We collected PBMCs from five healthy HLA-A2^+^ donors B–F to investigate the seven selected MUT peptides’ ability (MUC16-A9832D, SYNE1-V7402I, TP53-P190L, TP53-R267P, ABCA13-E1359Q, NFE2L2-D13N, and PCLO-E4090Q) to induce a T cell response. After three rounds stimulated by MUT peptides, peptide-specific T cells were collected and co-cultured with T2A2 cells loaded with MUT or WT peptides to detect IFN-γ release and lysis cytotoxicity.

**Figure 1:**
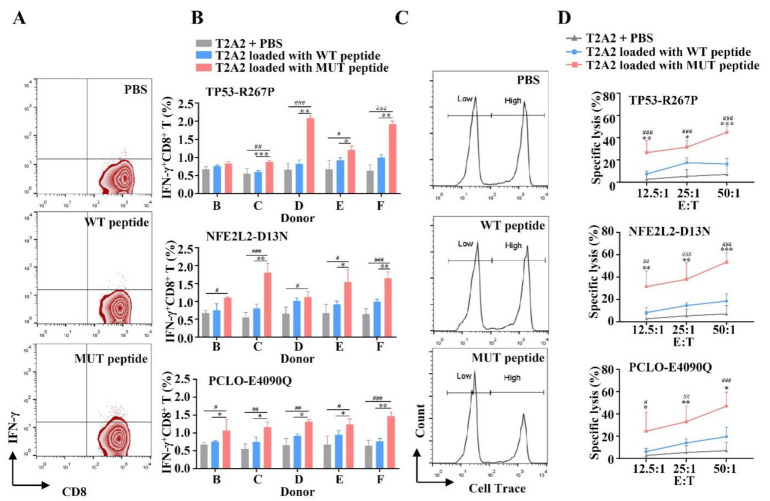
The immunogenicity of candidate mutant peptide-induced T cells to peptide-pulsing T2A2 cells in vitro. PBMCs isolated from five healthy HLA-A2^+^ donors (donors B–F) were induced by mature DCs pulsed by MUT peptides P53-R267P, NFE2L2-D13N, or PCLO-E4090Q (10 μg/mL) once a week for three weeks. After three rounds of stimulation, peptide-specific T cells were collected and co-cultured with T2A2 cells loaded with MUT or WT peptides and then were detected for IFN-γ release. (**A**) Representative zebra plots. (**B**) n = 3, three duplicate samples of each donor, and lysis cytotoxicity. (**C**) Typical histogram profiles. (**D**) n = 5. T2A2 + PBS group served as negative control. Statistical significance was determined by Student’s *t*-test. * *p* < 0.05, ** *p* < 0.01, *** *p* < 0.001 represented the significance of T2A2 cells loaded with MUT peptide group versus T2A2 cells loaded with WT peptide group; ^#^ *p* < 0.05, ^##^ *p* < 0.01, ^###^ *p* < 0.001 represented the significance of T2A2 cells loaded with MUT peptide group versus T2A2 + PBS group.

Results in Figure 1A,B and Figure S1A show that all seven of these MUT peptide-induced T cells could release IFN-γ, of which the IFN-γ release of each T2A2 loaded with MUT peptide from the TP53-R267P, NFE2L2-D13N, or PCLO-E4090Q group was higher than that of T2A2 unpulsed control group (Figure 1A) and was significantly different in at least 4/5 donors (^#^ *p* < 0.05, ^##^ *p* < 0.01, and ^###^ *p* < 0.001). To test whether the CTLs induced by MUT peptide were MUT peptide-specific, the CTLs induced were co-incubated with T2A2 cells loaded with MUT or WT peptides. The proportion of IFN-γ^+^ CD8^+^ T cells in T2A2 cells loaded with the MUT peptide group was higher than that of T2A2 cells loaded with the WT peptide group (Figure 1A,B) and was significantly different in at least in 3/5 donors (* p < 0.05, ** p < 0.01, and *** p < 0.001). These results show that T cells induced by MUT peptide can recognize MUT peptide-pulsing T2A2 cells in a MUT peptide-restricted manner. Thus, all seven of the MUT peptides are immunogenic, but the CTLs induced by MUC16-A9832D, SYNE1-V7402I, TP53-P190L, and ABCA13-E1359Q cannot effectively distinguish T2A2 cells loaded with WT or MUT peptides (Figure 1A,B and Figure S1A).

In the cytotoxicity assay, T cells induced by MUT peptides TP53-R267P, NFE2L2-D13N, or PCLO-E4090Q could efficiently lyse corresponding MUT peptide-pulsing T2A2 cells at series E/T ratio, while the cytotoxicity activity of those T cells to unpulsed T2A2 cells was extremely low (for TP53-R267P, *^###^ p* < 0.001, *^###^ p* < 0.001, and *^###^ p* < 0.001; for NFE2L2-D13N, *^##^ p* < 0.01, *^###^ p* < 0.001, and *^###^ p* < 0.001; for PCLO-E4090Q, ^*#*^ *p* < 0.05, *^##^ p* < 0.01, and ^*###*^ *p* < 0.001, at 12.5:1, 25:1, and 50:1, respectively). In addition, the lysis ratio of the WT group was very moderate compared with that of the MUT group (for TP53-R267P, ** *p* < 0.01, * *p* < 0.05, and *** *p* < 0.001; for NFE2L2-D13N, ** p < 0.01, ** p < 0.01, and *** p < 0.001; for PCLO-E4090Q, * p < 0.05, ** p < 0.01, and * p < 0.05, at 12.5:1, 25:1, and 50:1, respectively) (Figure 1C,D). These results indicate that T cells induced by MUT peptides TP53-R267P, NFE2L2-D13N, and PCLO-E4090Q showed potent cytotoxicity activity in a MUT peptide-restricted manner. In addition, CTLs induced by MUT peptides MUC16-A9832D, SYNE1-V7402I, TP53-P190L, or ABCA13-E1359Q also had a strong cytotoxicity activity. However, the lysis ratios of these CTLs on T2A2 cells loaded with MUT or WT peptides were similar. This result also indicates that CTLs stimulated by these four MUT peptides could not effectively distinguish T2A2 cells pulsed with WT or MUT peptides (Figure S1B).

Hence, MUT peptides TP53-R267P, NFE2L2-D13N, and PCLO-E4090Q are immunogenic, and the peptide-specific T cells they induced can recognize peptide-pulsing T2A2 cells in MUT a peptide-restricted way in vitro. MUT peptides MUC16-A9832D, SYNE1-V7402I, TP53-P190L, and ABCA13-E1359Q were excluded from further study.

### 3.3. MUT Peptide-Specific T Cells Can Recognize MUT Peptides Presented in Tumor CELL Line in an HLA-A2-Restricted and MUT Peptide-Specific Manner In Vitro

To figure out whether the MUT peptides TP53-R267P, NFE2L2-D13N, and PCLO-E4090Q were endogenously processed and presented by transfected tumor cells, and whether MUT peptide-specific CTLs could recognize these MUT peptides presented on the surface of transfected tumor cells, we constructed two minigenes (containing WT or MUT sequences) that encoded these three MUT peptides or their corresponding WT peptides (described in Supplemental Materials). These two minigenes were then transfected individually into tumor cell lines KYSE140 (HLA-A2^+^) and KYSE150 (HLA-A2^−^) to obtain KYSE140-WT (HLA-A2^+^, MUT peptide^−^), KYSE140-MUT (HLA-A2^+^, MUT peptide^+^), KYSE150-WT (HLA-A2^−^, MUT peptide^−^), and KYSE150-MUT (HLA-A2^−^, MUT peptide^+^) target cells. PBMCs were collected from five healthy HLA-A2^+^ donors G–K and induced by MUT peptides TP53-R267P, NFE2L2-D13N, and PCLO-E4090Q, respectively.

**Figure 2:**
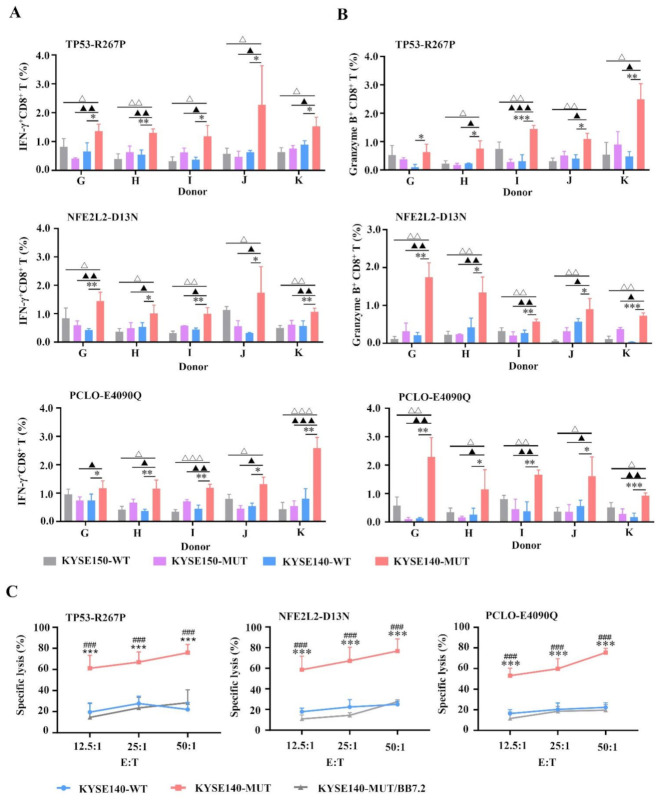
The IFN-γ release and KYSE tumor cell lines lysis of MUT peptide-induced T cells in vitro. PBMCs isolated from five healthy HLA-A2^+^ donors (donors G–K) were induced by mature DCs pulsed by MUT peptides P53-R267P, NFE2L2-D13N, or PCLO-E4090Q (10 μg/mL), respectively, once a week for three weeks. Two minigenes that encoded these three MUT peptides or their corresponding WT peptides were constructed and transfected individually into tumor cell lines KYSE140 (HLA-A2^+^) or KYSE150 (HLA-A2^−^). Transfected cells KYSE140-WT (HLA-A2^+^, MUT peptide^−^), KYSE140-MUT (HLA-A2^+^, MUT peptide^+^), KYSE150-WT (HLA-A2^−^, MUT peptide^−^), and KYSE150-MUT (HLA-A2^−^, MUT peptide^+^) were served as target cells to detect CTL response via IFN-γ secretion and granzyme B expression in the intracellular cytokine assay ((**A**,**B**), n = 3, three duplicate samples of each donor). The target cells in cytotoxicity assay are KYSE140-WT, KYSE140-MUT, and KYSE140-MUT/BB7.2 ((**C**), n = 5). Statistical significance was determined by Student’s *t*-test. In IFN-γ secretion and granzyme B expression assay, * *p* < 0.05, ** *p* < 0.01, *** *p* < 0.001 represented the significance of KYSE140-MUT group versus KYSE140-WT group; ^▲^ *p* < 0.05, ^▲▲^ *p* < 0.01, ^▲▲▲^ *p* < 0.001 represented the significance of KYSE140-MUT group versus KYSE150-MUT group; ^△^ *p* < 0.05, ^△△^ *p* < 0.01, ^△△△^ *p* < 0.001 represented the significance of KYSE140-MUT group versus KYSE150-WT group. In lysis cytotoxicity assay, ^###^ *p* < 0.001 represented the significance of KYSE140-MUT group versus KYSE140-MUT/BB7.2 group; *** *p* < 0.001 represented the significance of KYSE140-MUT group versus KYSE140-WT group.

In the intracellular cytokine assay, KYSE140-WT, KYSE140-MUT, KYSE150-WT, and KYSE150-MUT were served as target cells and co-incubated with each MUT peptide-induced T cell to detect CTL response via IFN-γ secretion and granzyme B expression. CTLs stimulated by the KYSE140-MUT group displayed a higher proportion of IFN-γ^+^ CD8^+^ T cells (for TP53-R267P, * *p* < 0.05 or ** *p* < 0.01; for NFE2L2-D13N, * *p* < 0.05 or ** *p* < 0.01; for PCLO-E4090Q, * *p* < 0.05 or ** *p* < 0.01 in five donors) and granzyme B^+^CD8^+^ T cells (for TP53-R267P, * *p* < 0.05 or ** *p* < 0.01 or *** *p* < 0.001; for NFE2L2-D13N, * *p* < 0.05 or ** *p* < 0.01 or *** *p* < 0.001; for PCLO-E4090Q, * *p* < 0.05 or ** *p* < 0.01 or *** *p* < 0.001 in five donors) compared with that of the KYSE140-WT (HLA-A2^+^, MUT peptide^−^) group; similar results were obtained when compared with that of the KYSE150-MUT (HLA-A2^−^, MUT peptide^+^) (^▲^ *p* < 0.05) or KYSE150-WT (HLA-A2^−^, MUT peptide^−^) (^△^ *p* < 0.05) group (Figure 2A,B). These results confirm that MUT peptides TP53-R267P, NFE2L2-D13N, and PCLO-E4090Q were endogenously processed and presented by transfected cells and that MUT peptide-induced CTLs specifically recognize MUT peptides presented on the surface of transfected cells. Moreover, MUT peptide-specific CTLs recognized target cells in an HLA-A2-restricted manner and could not recognize HLA-A2^−^ tumor cell lines (KYSE150-WT, KYSE150-MUT) if they presented MUT or WT peptide (Figure 2A,B). A cytotoxicity assay showed that CTLs induced by MUT peptides specifically lyse KYSE140-MUT rather than KYSE140-WT or KYSE140-MUT/BB7.2 (HLA-A2^−^, MUT peptide^+^) (Figure 2C) (^###^ *p* < 0.001, *** *p* < 0.001, respectively, for all these three peptides at 12.5:1, 25:1, and 50:1). These results further confirm that MUT peptides TP53-R267P, NFE2L2-D13N, and PCLO-E4090Q were endogenously processed, presented by transfected cells, and recognized target cells in a MUT peptide-specific and HLA-A2-restricted manner.

### 3.4. HLA-A2-Restricted and MUT Peptide-Specific CTL Responses Can Be Induced from HLA-A2.1/K^b^ Tg Mice In Vivo

To assess whether MUT peptides can stimulate MUT peptide-specific CTL response in vivo. HLA-A2.1/K^b^ Tg mice were used. After subcutaneous immunization of the HLA-A2.1/K^b^ Tg mice with MUT peptides at the base of the tail for 3 times, the lymphocytes from the spleen of immunized mice were re-stimulated with MUT peptides for another six days to detect the CTL response by intracellular cytokine assay and cytotoxicity assay.

**Figure 3:**
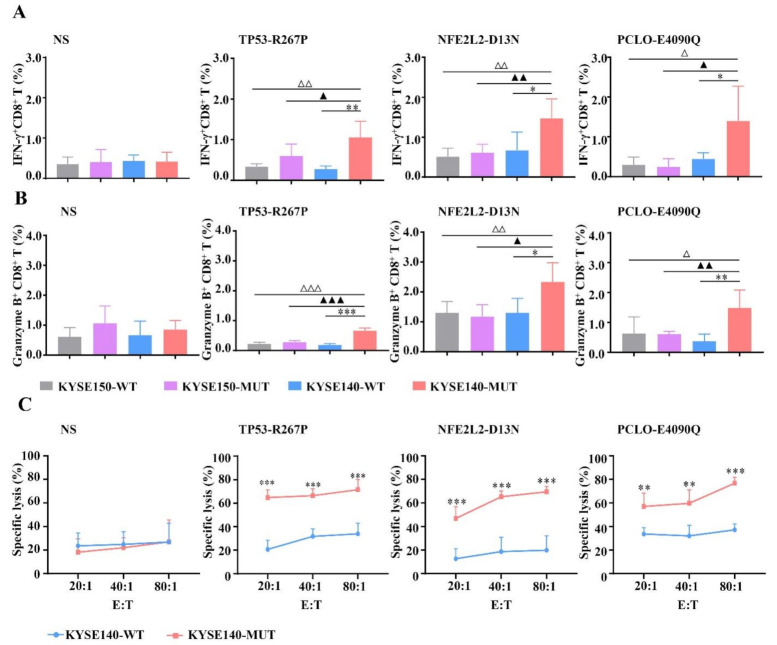
MUT peptide-specific and HLA-A2-restricted CTL responses in HLA-A2.1/K^b^ transgenic mice. An amount of 100 μg/mouse TP53-R267P, NFE2L2-D13N, PCLO-E4090Q, or normal saline (NS) was respectively injected s.c. at the end of the tail (n = 5 per group) with CpG ODN 1826 (30 μg/mouse) once a week for three weeks. Then the spleen lymphocytes were re-stimulated with MUT peptides and co-cultured with respective target cells to detect the CTL response via IFN-γ secretion and granzyme B expression in the intracellular cytokine assay (**A**,**B**) and cytotoxicity assay (**C**). Transfected cells KYSE140-WT (HLA-A2^+^, MUT peptide^−^), KYSE140-MUT (HLA-A2^+^, MUT peptide^+^), KYSE150-WT (HLA-A2^−^, MUT peptide^−^), and KYSE150-MUT (HLA-A2^−^, MUT peptide^+^) were served as stimulator cells and target cells. Statistical significance was determined by Student’s *t*-test. In IFN-γ secretion and granzyme B expression assay, * *p* < 0.05, ** *p* < 0.01, *** *p* < 0.001 represented the significance of KYSE140-MUT group versus KYSE140-WT group; ^▲^ *p* < 0.05, ^▲▲^ *p* < 0.01, ^▲▲▲^ *p* < 0.001 represented the significance of KYSE140-MUT group versus KYSE150-MUT group; ^△^ *p* < 0.05, ^△△^ *p* < 0.01, ^△△△^ *p* < 0.001 represented the significance of KYSE140-MUT group versus KYSE150-WT group. In lysis cytotoxicity assay, ** *p* < 0.01, *** *p* < 0.001 represented the significance of KYSE140-MUT group versus KYSE140-WT group.

KYSE140-WT, KYSE140-MUT, KYSE150-WT, and KYSE150-MUT were served as target cells and co-incubated with these induced CTLs to detect CTL response via IFN-γ secretion and granzyme B expression in intracellular cytokine assay. We found that CTLs induced by MUT peptides recognized KYSE140-MUT but no other control target cells (KYSE140-WT, KYSE150-WT, and KYSE150-MUT), in an HLA-A2-restricted and MUT peptide-specific manner in vivo (* *p* < 0.05, ^▲^ *p* < 0.05, ^△^ *p* < 0.05) (Figure 3A,B). In cytotoxicity assay, CTLs induced by MUT peptides displayed higher lysis rates in the KYSE140-MUT group compared with the KYSE140-WT group (for TP53-R267P and NFE2L2-D13N, *** *p* < 0.01, *** *p* < 0.01, *** *p* < 0.01; for PCLO-E4090Q, ** *p* < 0.01, ** *p* < 0.01, *** *p* < 0.001, at 20:1, 40:1, and 80:1, respectively) (Figure 3C).

Taken together, these findings indicate that MUT peptides can induce HLA-A2-restricted and MUT peptide-specific CTL responses in vivo.

### 3.5. Enhanced CTL Responses by WH-MUT Peptides Induced from HLA-A2.1/K^b^ Tg Mice

To enhance the MUT peptide-specific CTL response, these MUT peptides were individually coupled to peptide WH selected by our lab in a previous study and used as a powerful epitope carrier of Clec9a^+^DC to promote peptide-specific CTL response [16]. After subcutaneous immunization of the HLA-A2.1/K^b^ Tg mice with WH-MUT peptides at the base of the tail for 3 times, the lymphocytes from the spleen of immunized mice were re-stimulated with MUT peptides for another six days to detect the CTL response by intracellular cytokine assay and cytotoxicity assay.

**Figure 4:**
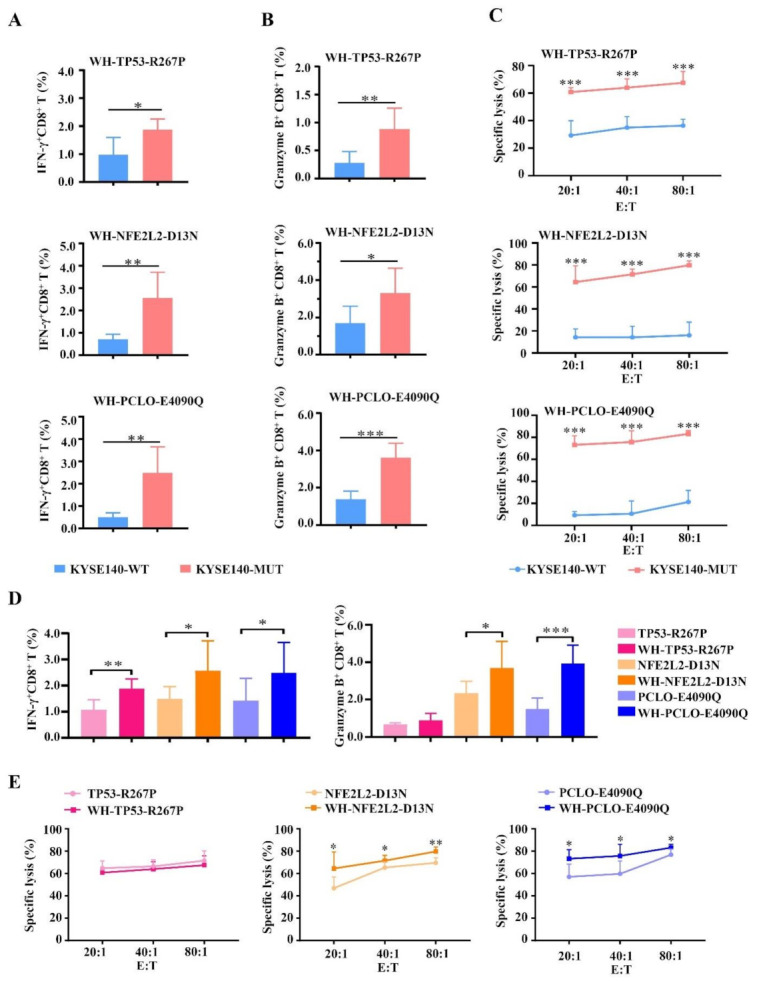
Enhanced MUT peptide-specific and HLA-A2-restricted CTL responses in HLA-A2.1/K^b^ transgenic mice. An amount of 100 μg/mouse WH-TP53-R267P, WH-NFE2L2-D13N, WH-PCLO-E4090Q, or normal saline (NS) was respectively injected s.c. at the end of the tail (n = 5 per group) with CpG ODN 1826 (30 μg/mouse) once a week for three weeks. Then the spleen lymphocytes were re-stimulated with MUT peptides and co-cultured with respective target cells to detect the CTL response via IFN-γ secretion (**A**) and granzyme B expression (**B**) in the intracellular cytokine assay and cytotoxicity assay (**C**). When the KYSE140-MUT served as target cells, the comparation of CTLs’ IFN-γ secretion and granzyme B expression (**D**) and lysis cytotoxicity (**E**) between MUT peptide and WH-MUT verified the improved immunogenicity of MUT peptide coupling with WH peptide. Transfected cells KYSE140-WT (HLA-A2^+^, MUT peptide^−^), KYSE140-MUT (HLA-A2^+^, MUT peptide^+^), KYSE150-WT (HLA-A2^−^, MUT peptide^−^), and KYSE150-MUT (HLA-A2^−^, MUT peptide^+^) were served as stimulator cells and target cells. Statistical significance was determined by Student’s *t*-test. In IFN-γ secretion and granzyme B expression assay, * *p* < 0.05, ** *p* < 0.01, *** *p* < 0.001 represented the significance of KYSE140-MUT group versus KYSE140-WT group. In lysis cytotoxicity assay, *** *p* < 0.001 represented the significance of KYSE140-MUT group versus KYSE140-WT group. In D–E, * p < 0.05, ** *p* < 0.01, *** *p* < 0.001 represented the significance of WH-MUT peptide group versus MUT peptide group.

KYSE140-WT and KYSE140-MUT were served as target cells and co-incubated with these induced CTLs to detect CTL response via IFN-γ secretion and granzyme B expression in intracellular cytokine assay. CTLs induced by WH-MUT peptides exhibited notable responses in the HLA-A2-restricted and MUT peptide-specific manner in vivo (Figure 4). Remarkably, we found that CTLs induced by these WH-MUT peptides exhibited stronger IFN-γ secretion (WH-TP53-R267P: 1.9%; WH-NFE2L2-D13N: 2.6%; and WH-PCLO-E4090Q: 2.5%) than their corresponding MUT peptides (TP53-R267P: 1.1%; NFE2L2-D13N: 1.5%; and WH-PCLO-E4090Q: 1.4%) in KYSE140-MUT groups (Figure 4D). The expressions of granzyme B also were potently triggered by WH-MUT peptides (TP53-R267P: 0.7%; WH-TP53-R267P: 0.9%; NFE2L2-D13N: 2.3%; WH-NFE2L2-D13N: 3.7%; PCLO-E4090Q: 1.5%; and WH-PCLO-E4090Q: 3.9%) in KYSE140-MUT groups (Figure 4D). Except WH-TP53-R267P, CTLs induced by WH-MUT peptides displayed higher lysis rates in the KYSE140-MUT group (WH-TP53-R267P: 67.5%; WH-NFE2L2-D13N: 79.7%; and WH-PCLO-E4090Q: 83.2%) than MUT-peptide (TP53-R267P: 71.5%; NFE2L2-D13N: 69.5%; and PCLO-E4090Q: 76.7%) at an E/T of 80:1 (Figure 4E) in cytotoxicity assay.

All these findings show that WH-MUT peptides can induce HLA-A2-restricted and MUT peptide-specific CTL responses in vivo and that the immunogenicity of these MUT peptides was improved after coupling with peptide WH.

### 3.6. The Docking Results of the Candidate Peptide and HLA-A*0201 Molecule

**Figure 5:**
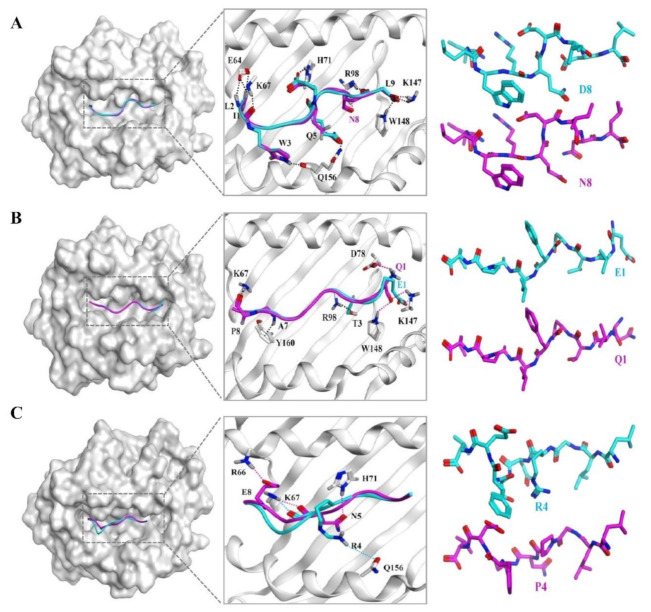
The possible structural models of the candidate peptide and HLA-A*0201 molecule. The structures of the WT peptides and MUT peptides were predicted by PEP-Fold. WT peptide (blue, (**A**): NFE2L2-WT; (**B**): PCLO-WT; (**C**): TP53-WT) or MUT peptide (magenta, (**A**): NFE2L2-D13N; (**B**): PCLO-E4090Q; (**C**): TP53-R267P) were docked with HLA-A*0201 molecule (gray) (PDB ID: 5YXN) by MOE (Molecular Operating Environment software). The binding sites of the peptides to HLA-A*0201 molecules are labelled.

In our study, we found that MUT peptides NFE2L2-D13N, PCLO-E4090Q, and TP53-R267P recognized MUT peptides in an HLA-A2-restricted as well as in a MUT peptide-specific manner in vitro and in vivo. Structures of these three WT (blue)/MUT (magenta) peptides were docked in the binding groove of HLA-A*0201 using MOE. The structural models of NFE2L2-D13N (Figure 5A) show that position 8 forms one new hydrogen bond with position 98 (R) of the HLA-A*0201 molecule, compared with the WT peptide NFE2L2-D13. The structural models of PCLO-E4090Q (Figure 5B) show that position 1 forms two new hydrogen bonds and one ionic bond with position 148 (W) and 78 (D) of the HLA-A*0201 molecule, respectively, compared with the WT peptide PCLO-E4090. The structural models of TP53-R267P (Figure 5C) show that position 4 reduces by one hydrogen bond with position 156 (Q) of the HLA-A*0201 molecule compared with the WT peptide TP53-R267. Otherwise, the structural models of ABCA13-E1359Q, MUC16-A9832D, SYNE1-V7402I, and TP53-P190L (Figure S2) show that the mutation site at position 2 or 3 could not distinguish the WT and MUT peptide-specifically, regardless of whether forming new bond.

## 4. Discussion

Despite the numerous advances that have been made in the diagnosis, treatment, and pathogenesis of esophageal squamous cell carcinoma, the overall 5-year survival rate has been hovering around 15–25%, and the morbidity and mortality are still increasing [2,32,33]. Surgery remains the first treatment option, but 70% of ESCC patients are diagnosed in the advanced stage and have lost the opportunity for surgery, and the survival of most metastatic patients is only 7 to 10 months [34]. Therefore, the development of new strategies is particularly important for the treatment of ESCC.

In this study, we predicted HLA-A2-restricted mutant peptides derived from the most significantly mutant antigens of ESCC according to three recent publications [13,14,15]. Seven of the predicted mutant peptides exhibited vigorous binding affinity and formed a stable peptide/HLA complex with HLA-A2 molecules. Three MUT peptides (TP53-R267P, NFE2L2-D13N, and PCLO-E4090Q) of the seven were immunogenic, and the CTLs they induced were MUT peptide-specific. The minigene approach was carried out to examine the immune competence of the three MUT peptides according to the report of Steven A. Rosenberg [35]. The results confirmed that CTLs induced by MUT peptides TP53-R267P, NFE2L2-D13N, and PCLO-E4090Q recognize MUT peptides presented on transfected cells in an HLA-A2-restricted as well as in a MUT peptide-specific manner in vitro and in vivo. CTL response was improved by coupling MUT peptides to peptide WH, a peptide delivery carrier targeting Clec9a^+^ DCs [16,36]. The docking structural models of the candidate peptides/HLA-A*0201 complex showed that the immunogenicity of the MUT peptides could be influenced by the newly formed bond between the mutant position of the MUT peptide with the HLA-A*0201. Our results suggest that position 1 or 8 may play an important role in the recognition of WT/MUT peptides of the MUT peptide-induced CTLs. This result may lay a foundation for the optimization of prediction software based on mutant epitope peptides that can accurately screen the candidate mutant peptide. In addition, the results in Figure 2 show that the MUT peptides could be naturally processed and presented in transfected cells; thus, it will be very important to show that endogenously produced MUT peptides in primary ESCC cells can be recognized by MUT-specific CTL in future studies.

Neoantigens are the tumor-specific antigens that are not expressed in normal tissues, and they have been considered as one of the most ideal targets for inducing powerful antitumor immune responses and determining the fate of tumor patients, with the promise of providing more effective and more generalizable treatments than current therapies [37,38]. Numerous clinical trials based on mutant peptide have provided a strong rationale for tumor treatment, especially in melanoma. Thus, the discovery of mutant epitopes derived from mutant antigens becomes the key part in immunotherapy for cancer. Recent research has highlighted the attractive power of next-generation sequencing of cancer in identification of the CTL epitope from mutant antigens of a tumor. Eric Tran and Steven A. Rosenberg had demonstrated the immunogenicity of somatic mutations derived from gastrointestinal cancers using a next-generation sequencing method combined with high-throughput immunologic screening [39]. Though many neoantigens from different patients’ tumors were selected, there are currently no reported results of immune checkpoint inhibitors or mutant peptides for clinical treatment in ESCC.

Cancer immunotherapy based on mutant peptides is one of the most promising candidates. Massive data indicated that there is a significant correlation between tumor mutant antigen burden and objective response rate of checkpoint blockade immunotherapy. These data provide a strong rationale for combining the approach based on mutant peptides with checkpoint blockade immunotherapy. Eric Tran et al. had identified a CD4^+^ T cell epitope ERBB2IP-E805G derived from a metastatic cholangiocarcinoma patient; they found that mutation-reactive CD4^+^ Th1 cells could be used to mediate the regression of the tumor [40]. Keskin, D.B et al. also found that neoantigen-targeting vaccines could induce neoantigen-specific CD4^+^ and CD8^+^ T cell responses and alter the immune microenvironment of glioblastoma [41]. Compared with tumor-associated antigen, neoantigens are as specific as a “sniper”, and this feature makes them less harmful to the normal tissues of patients. Our research focused on the HLA-A2 haplotype, which is expressed among 37.7% of the Chinese population. This strategy will allow as many patients as possible to benefit from the identified neoantigens. We also analyzed the missense mutation rate of these three MUT peptides using the COSMIC database; the results show that the missense mutation rate of TP53-R267P is 1.02% (155/15134) in tumor patients. The missense mutation rate of PCLO-E4090Q is 1.85% (3/162) in esophageal carcinoma. Additionally, the missense mutation rate of NFE2L2-D13N is 1.73% (13/751) in tumor patients, including 3.3% (9/272) in lung cancer, 2.02% (2/99) in liver cancer, and 1.01% (1/99) in esophageal carcinoma. Thus, the peptides identified in our study have certain patient coverage and potential clinical application prospects. In addition, spontaneous immune recognition of neoantigen is clinically inefficient. We coupled MUT peptides with peptide WH, which is a delivery carrier targeting Clec9a^+^ DCs. The CTL response was strikingly promoted. This strategy could be used for other tumor antigens to enhance the T cell response. Other strategies were also exploited to use the neoepitopes for the treatment of tumor patients. Ugur Sahin implemented an RNA-based poly-neoepitope approach to induce T cells that could specifically lyse the autologous melanoma cells. After vaccination, the rate of metastasis and recurrence was significantly reduced, and patients underwent a sustained progression-free survival [42]. Gal Cafri et al. also found that mRNA vaccines encoding neoepitopes could induce neoantigen-specific T cell response in patients with metastatic gastrointestinal cancer [43]. These RNA vaccines often contained several neoepitopes, which greatly broaden patient coverage. Furthermore, James J. Moon et al. employed nanodiscs coupled with a neoepitopes vaccine to stimulate strong CD8^+^ CTL responses and found that the nanodiscs released 47-fold greater frequencies of neoantigen-specific CTLs than soluble vaccines [44]. All of these strategies take advantage of the immunogenicity of neoantigens and lay the foundation for subsequent clinical application of the neoepitopes identified in this study.

In conclusion, three HLA-A2-restricted immunogenic mutant peptide-derived neoepitopes from mutant antigen of ESCC were first identified. All of these three neoepitopes can induce an HLA-A2-restricted and a MUT peptide-specific CD8^+^ T cell response both in vitro and in vivo, and the CD8^+^ T cell induced by three neoepitopes can be enhanced by coupling MUT peptides to peptide WH. The neoepitopes identified will provide candidate neoepitopes for the development of tumor vaccines and immunotherapy for patients with ESCC.

## Figures and Tables

**Table 1:** Prediction scores of the mutant peptides derived from mutant antigens in ESCC.

Gene	Position	Sequence	Scores		
			IEDB	NetCTL	SYFPEITHI
ABCA13	D1303H	NLHSINDFL	2	1.0131	20
	E1359Q	IL**Q**DGFLYV	0.2	1.3979	26
DNAH5	S3587Y	GLPNDDL**Y**I	3.3	0.9645	20
	D4110N	FM**N**ELMD I	0.6	1.2466	21
	L4406H	RMQRVLS**H**V	1.6	0.9677	22
	M4495T	FLTA**T**RQEI	0.8	0.9167	20
KMT2D	R4198Q	QL**Q**AQLQGV	0.9	0.9111	25
	F4722L	ILGEEAPR**L**	2.5	0.8820	25
LRP1B	C2479Y	**Y**LLTPNGRV	1.2	1.0572	25
	R3362L	G**L**FQCGTGL	2.4	1.0649	23
	P3707L	A**L**DMCVKFL	1.6	0.9640	25
LRP2	D1744Y	CLRD**Y**QPFL	1.2	1.2251	23
MUC16	A4579D	SMGDAL**D**SI	2	1.1824	25
	Q5024H	LMSRIP**H**DV	1.8	0.8792	21
	S5361F	SIPS**F**PLPV	1.8	1.1484	22
	A9832D	VL**D**DSETTI	2.1	1.1934	22
MUC17	A2414V	TMPVVSSE**V**	2.3	0.9571	20
	T3809M	T**M**SERSTLL	2.4	1.1026	20
NEB	D3282V	VISDYKYK**V**	1.3	1.1003	24
NFE2L2	D13N	ILWRQDI**N**L	1.6	1.0632	23
	I28T	ILWRQD**T**DL	3.6	0.7755	23
NOTCH1	G1995V	RMHD**V**TTPL	0.8	1.4064	21
	S2202F	GMLSPVD**F**L	1.8	1.1812	26
PCDH15	S628L	T**L**TATVNIV	1.7	0.8437	24
PCLO	E4090Q	**Q**VTDFLAPL	3.8	1.0399	21
SYNE1	A65S	KLL**S**LLEVL	0.8	1.2351	28
	V7402I	FL**I**QTEQKL	0.5	1.0359	25
	S7628Y	**Y**LPDHHEEL	0.8	1.3406	25
	N4342I	T**I**LEELNVV	3	0.9190	27
TP53	C135F	ALNKMF**F**QL	1.2	1.2333	23
	P190L	A**L**PQHLIRV	1	1.2385	26
	G244V	YMCNSSCM**V**	0.5	1.0763	20
	G266A	LL**A**RNSFEV	0.4	1.2765	26
	R267P	LLG**P**NSFEV	0.4	1.2604	26
	V272L	LLGRNSFE**L**	1.6	0.9715	24

**Table 2:** The data of ESI-MS and the HLA-A*0201 binding affinity and stability of the mutant peptides.

Gene	Position	ESI-MS [M+H]^+^		FI ^a^	DC _50_^b^
		Calculated	Observed		
ABCA13	E1359Q	1067.25	1067.87	1.56	>6 h
KMT2D	R4198Q	984.12	985.29	1.12	<2 h
MUC16	A4579D	908	909.05	1.35	<2 h
	A9832D	992.05	993.04	3.56	>6 h
	S5361F	956.15	957.03	2.25	<2 h
MUC17	A2414V	948.11	948.80	1.54	<4 h
NFE2L2	D13N	1170.38	1171.03	1.67	>6 h
PCLO	E4090Q	1003.16	1003.87	1.85	>4 h
SYNE1	S7628Y	1152.23	1153.55	1.67	<2 h
	N4342I	1029.2	1030.16	1.81	<4 h
	V7402I	1119.33	1120.6	1.27	>6 h
TP53	P190L	1046.28	1047.51	1.26	>4 h
	R267P	975.11	976.26	1.81	>6 h
COX-2	321–329	999.6	1000.3	2.12	>6 h

^a^ FI = (MFI of the given peptide—MFI of the PBS control group without peptides)/MFI of the PBS control group without peptides. ^b^ DC_50_ was calculated as follow: [MFI of 0 h—MFI of (2, 4, or 6 h)]/MFI of 0 h × 100%.

## Supplementary Materials

The following are available online at https://www.mdpi.com/article/10.3390/vaccines9101118/s1, Figure S1: The immunogenicity of the rest mutant peptides induced T cells to peptide-pulsing T2A2 cells in vitro, Figure S2: The possible structural models of the other MUT peptide and HLA-A*0201 molecule, Table S1: Data of ESI-MS and the HLA-A*02 binding affinity and stability of other mutant peptides.
