# Supplementary material for: Identification of HLA-A2-Restricted Mutant Epitopes from Neoantigens of Esophageal Squamous Cell Carcinoma
Source: Vaccines (Basel). 2021 Oct 1;9(10):1118. doi: 10.3390/vaccines9101118 (PMC8541546; doi:10.3390/vaccines9101118)
Supplement: Supplementary file 1 [file vaccines-09-01118-s001.zip › vaccines-1352736-supplementary.pdf]

Supplementary Methods and Materials

The construction of minigene and transfection of tumor cell lines

Two 51 aa long minigenes which encompassing the mutant peptides TP53-R267P, NFE2L2-D13N, PCLO-E4090Q or the corresponding wild type sequence, flank on each side by 8 amino acids of the WT sequence, were cloned using EcoRI and BamHI restriction sites in the lentiviral vector pLVX-IRES-ZsGreen1(Ampicillin) upstream of an IRES sequence preceding a GFP tag. Sequence of each minigene contain a 5’Kozak (GCCACC) sequence, a 5’ START codon (ATG) and a 3’ STOP codon (TGA):

Minigene of MUT peptides TP53-R267P, NFE2L2-D13N, PCLO-E4090Q
gac tcc agt ggt aat cta ctg gga cgg aac agc ttt gag gtg cgt gtt tgt gac ata ctt tgg agg caa gat ata
D S S G N L L G R N S F E V R V C D I L W R Q D I
gat ctt gga gta agt cga gaa gta ttt acc act gag aca cgc cgg tct caa gaa gtg aca gat ttc cta gca cct tta
D L G V S R E V F T T E T R R S Q E V T D F L A P L

Minigene of WT peptides TP53-R267P, NFE2L2-D13N, PCLO-E4090Q
gac tcc agt ggt aat cta ctg gga ccg aac agc ttt gag gtg cgt gtt tgt gac ata ctt tgg agg caa gat ata
D S S G N L L G R N S F E V R V C D I L W R Q D I
aat ctt gga gta agt cga gaa gta ttt acc act gag aca cgc cgg tct caa caa gtg aca gat ttc cta gca cct tta
D L G V S R E V F T T E T R R S Q E V T D F L A P L

Each lentivirus vector was produced upon HEK293-T packaging cells and then were respectively transfected into KYSE140 (HLA-A2$^+$) and KYSE150 (HLA-A2$^-$) tumor cell lines to get KYSE140-MUT (HLA-A2$^+$, MUT peptide$^+$), KYSE140-WT (HLA-A2$^+$, MUT peptide$^-$), KYSE150-WT (HLA-A2$^-$, MUT peptide$^-$) and KYSE150-MUT (HLA-A2$^-$, MUT peptide$^+$) cell lines.

Supplementary Tables

Table S1 Data of ESI-MS and the HLA-A*02 binding affinity and stability of other mutant peptides

Gene	Position	Peptide	ESI-MS[M+H]$^+$		FI[a]	DC_{50}[b]
			Calculated	Observed		
ABCA13	D1303H	NL**H**SINDFL	1072.19	1072.85	0.24	Nd[c]
DNAH5	S3587Y	GLPNDDL**Y**I	1019.12	1020.1	0.68	Nd[c]
	D4110N	FM**N**ELMDII	1025.38	1126.54	0.38	Nd[c]
	L4406H	RMQRVLS**H**V	1125.36	1126.30	0.30	Nd[c]
	M4495T	FLTA**T**RQEI	1078.23	1079.22	0.21	Nd[c]
KMT2D	F4722L	ILGEEAPR**L**	997.16	998.31	0.29	Nd[c]
LRP1B	C2479Y	**Y**LLTPNGRV	1032.21	1033.31	0.39	Nd[c]
	R3362L	G**L**FQCGTGL	895.05	895.83	0.09	Nd[c]
	P3707L	A**L**DMCVKFL	1039.33	1040.11	0.37	Nd[c]
LRP2	D1744Y	CLRD**Y**QPFL	1154.35	1154.92	0.20	Nd[c]
MUC16	Q5024H	LMSRIP**H**DV	1067.28	1068.41	0.14	Nd[c]
MUC17	T3809M	T**M**SERSTLL	1037.22	1037.81	0.12	Nd[c]
NEB	D3282V	VISDYKYK**V**	1114.31	1115.01	0.09	Nd[c]
NFE2L2	I28T	ILWRQD**T**DL	1159.31	1159.98	0.79	Nd[c]
NOTCH1	G1995V	RMHD**V**TTPL	1069.25	1070.42	0.42	Nd[c]
	S2202F	GMLSPVD**F**L	978.18	979.24	0.13	Nd[c]
PCDH15	S628L	T**L**TATVNIV	931.1	931.83	0.43	Nd[c]
SYNE1	A65S	KLL**S**LLEVL	1027.31	1028.46	0.08	Nd[c]
TP53	C135F	ALNKMF**F**QL	1111.37	1112.57	0.12	Nd[c]
	G244V	YMCNSSCM**V**	1037.27	1038.17	0.49	Nd[c]
	G266A	LL**A**RNSFEV	1048.21	1049.41	0.16	Nd[c]
	V272L	LLGRNSFE**L**	1048.21	1049.44	0.53	Nd[c]

[a]FI= (MFI of the given peptide- MFI of the PBS control group without peptides)/ MFI of the PBS control group without peptides.

[b]DC_{50} was calculated as follow: [MFI of 0 h-MFI of (2, 4 or 6 h)]/MFI of 0 h × 100%

[c] Not determined.

Supplementary Figure

Fig S1

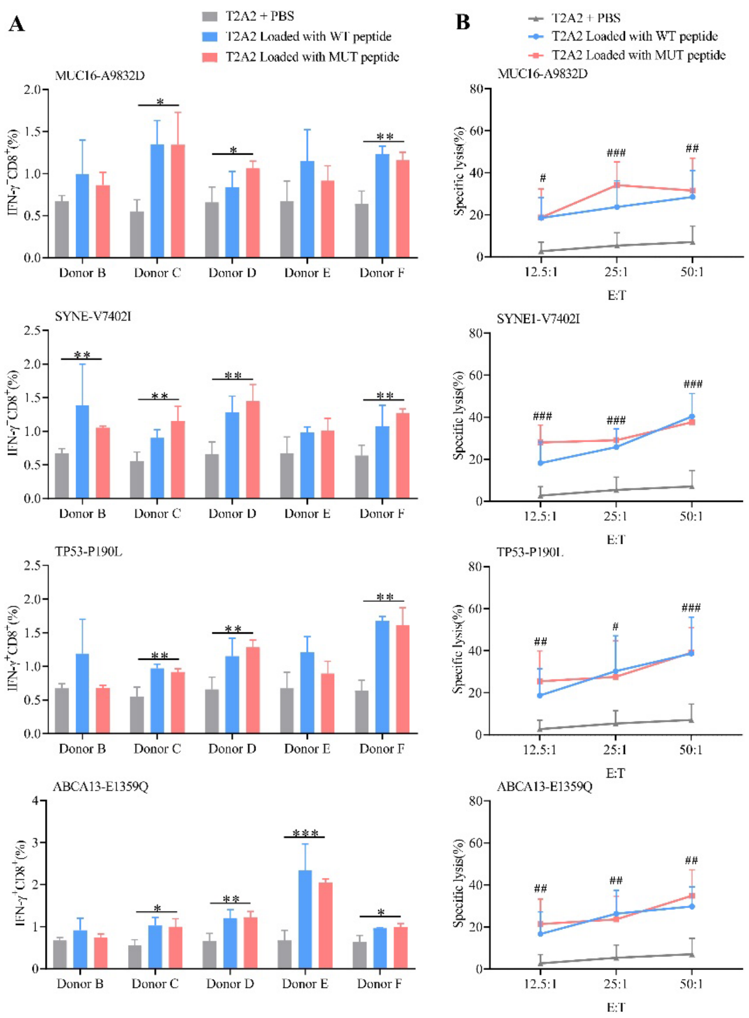

Figure S1. The immunogenicity of the rest mutant peptides induced T cells to peptide-pulsing T2A2 cells *in vitro*. PBMCs isolated from five healthy HLA-A2^{+} donors (donor B-F) were induced by mature DCs pulsed by MUT peptide MUC16-A9832D, SYNE1-V7402I, TP53-P190L or ABCA13-E1359Q (10 μg/mL) once a week**.** After three rounds stimulated by MUT peptides, CTLs were collected and co-cultured with T2A2 cells loaded with MUT or WT peptides and then were detected for IFN-γ release (**A**, n = 3) and lysis cytotoxicity (**B**, n = 5). T2A2 + PBS cells group severed as negative control. Statistical significance was determined by Student's t-test. $^{*}p < 0.05$, $^{**}p < 0.01$, $^{***}p < 0.001$ represented the significances of T2A2 cells loaded with MUT peptide group versus T2A2 cells loaded with WT peptide group, $^{\#}p < 0.05$, $^{\#\#}p < 0.01$, $^{\#\#\#}p < 0.001$ represented the significances of T2A2 cells loaded with MUT peptide group versus T2A2 + PBS cells group.

Fig S2

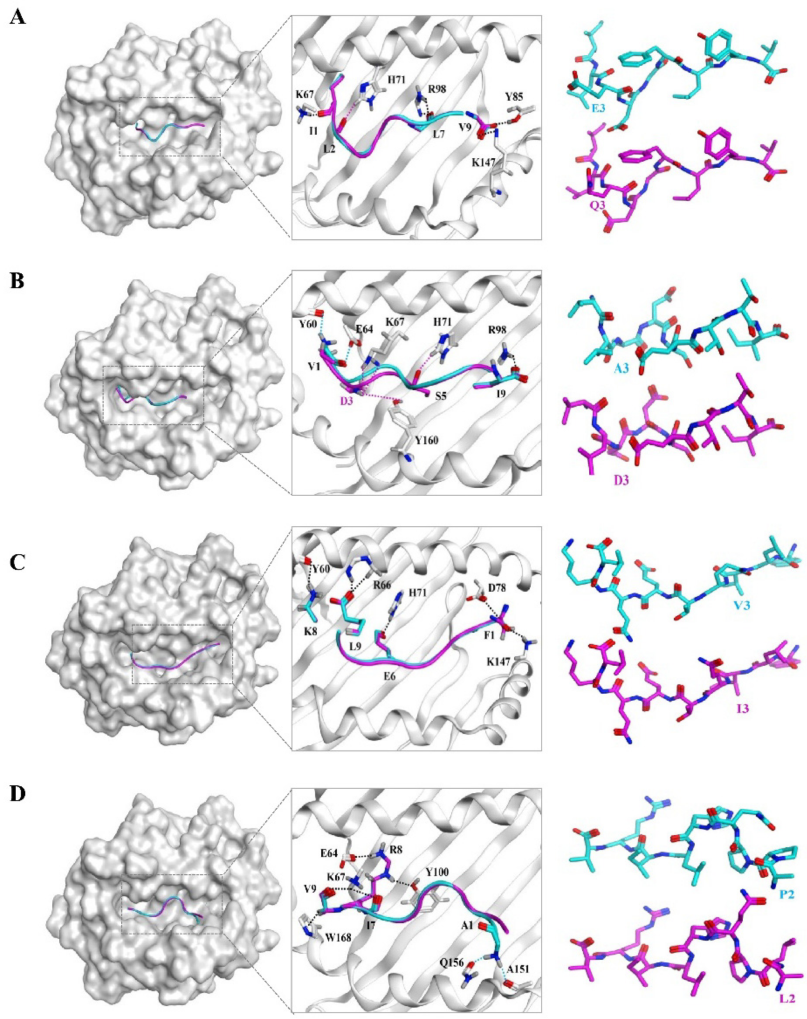

Figure S2. The possible structural models of the other MUT peptide and HLA-A*0201 molecule. The structures of the WT peptides and MUT peptides was predicted by PEP-Fold. WT Peptide (blue, **A**: ABCA13-WT; **B**: MUC16-WT; **C**: SYNE1-WT; **D**: TP53-WT) or MUT peptide (magenta, **A**: ABCA13-E1359Q; **B**: MUC16-A9832D; **C**: SYNE1-V7402I; **D**: TP53-P190L) was docked with HLA-A*0201 molecule (gray) (PDB ID: 5YXN) by MOE (Molecular Operating Environment software). The binding sites of the peptides to HLA-A*0201 molecules were labeled.
